# Supplementary material for: Causal Relationship between Aging and Anorexia Nervosa: A White-Matter-Microstructure-Mediated Mendelian Randomization Analysis
Source: Biomedicines. 2024 Aug 16;12(8):1874. doi: 10.3390/biomedicines12081874 (PMC11351342; doi:10.3390/biomedicines12081874)
Supplement: Supplementary file 1 [file biomedicines-12-01874-s001.zip › Supplementary Figure/Figure S4/Singe SNP plot of MR analysis for TL to ubm-b-1707.pdf]

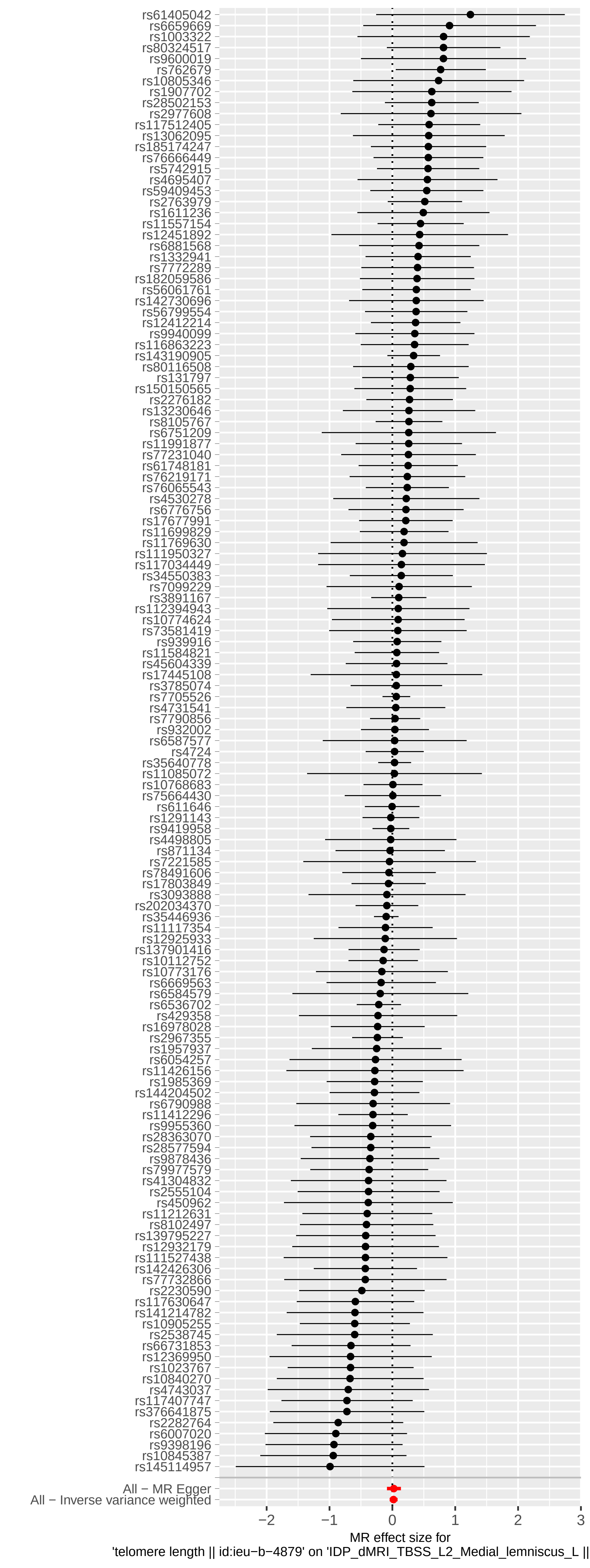
rs61405042
rs6659669
rs1003322
rs80324517
rs9600019
rs762679
rs10805346
rs1907702
rs28502153
rs2977608
rs117512405
rs13062095
rs185174247
rs76666449
rs5742915
rs4695407
rs59409453
rs2763979
rs1611236
rs11557154
rs12451892
rs6881568
rs1332941
rs7772289
rs182059586
rs56061761
rs142730696
rs56799554
rs12412214
rs9940099
rs116863223
rs143190905
rs80116508
rs131797
rs150150565
rs2276182
rs13230646
rs8105767
rs6751209
rs11991877
rs77231040
rs61748181
rs76219171
rs76065543
rs4530278
rs6776756
rs17677991
rs11699829
rs11769630
rs111950327
rs117034449
rs34550383
rs7099229
rs3891167
rs112394943
rs10774624
rs73581419
rs939916
rs11584821
rs45604339
rs17445108
rs3785074
rs7705526
rs4731541
rs7790856
rs932002
rs6587577
rs4724
rs35640778
rs11085072
rs10768683
rs75664430
rs611646
rs1291143
rs9419958
rs4498805
rs871134
rs7221585
rs78491606
rs17803849
rs3093888
rs202034370
rs35446936
rs11117354
rs12925933
rs137901416
rs10112752
rs10773176
rs6669563
rs6584579
rs6536702
rs429358
rs16978028
rs2967355
rs1957937
rs6054257
rs11426156
rs1985369
rs144204502
rs6790988
rs11412296
rs9955360
rs28363070
rs28577594
rs9878436
rs79977579
rs41304832
rs2555104
rs450962
rs11212631
rs8102497
rs139795227
rs12932179
rs111527438
rs142426306
rs77732866
rs2230590
rs117630647
rs141214782
rs10905255
rs2538745
rs66731853
rs12369950
rs1023767
rs10840270
rs4743037
rs117407747
rs376641875
rs2282764
rs6007020
rs9398196
rs10845387
rs145114957
All − MR Egger
All − Inverse variance weighted
−2
−1
0
1
2
3
MR effect size for
'telomere length || id:ieu−b−4879' on 'IDP_dMRI_TBSS_L2_Medial_lemniscus_L ||
